# Bank Vole Prion Protein As an Apparently Universal Substrate for RT-QuIC-Based Detection and Discrimination of Prion Strains

**DOI:** 10.1371/journal.ppat.1004983

**Published:** 2015-06-18

**Authors:** Christina D. Orrú, Bradley R. Groveman, Lynne D. Raymond, Andrew G. Hughson, Romolo Nonno, Wenquan Zou, Bernardino Ghetti, Pierluigi Gambetti, Byron Caughey

**Affiliations:** 1 Laboratory of Persistent Viral Diseases, Rocky Mountain Laboratories, National Institute of Allergy and Infectious Diseases (NIAID), National Institutes of Health (NIH), Hamilton, Montana, United States of America; 2 Department of Veterinary Public Health and Food Safety, Istituto Superiore di Sanità, Rome, Italy; 3 Department of Pathology, Case Western Reserve University, Cleveland, Ohio, United States of America; 4 Department of Pathology and Laboratory Medicine, Indiana University School of Medicine, Indianapolis, Indiana, United States of America; Dartmouth Medical School, UNITED STATES

## Abstract

Prions propagate as multiple strains in a wide variety of mammalian species. The detection of all such strains by a single ultrasensitive assay such as Real Time Quaking-induced Conversion (RT-QuIC) would facilitate prion disease diagnosis, surveillance and research. Previous studies have shown that bank voles, and transgenic mice expressing bank vole prion protein, are susceptible to most, if not all, types of prions. Here we show that bacterially expressed recombinant bank vole prion protein (residues 23-230) is an effective substrate for the sensitive RT-QuIC detection of all of the different prion types that we have tested so far – a total of 28 from humans, cattle, sheep, cervids and rodents, including several that have previously been undetectable by RT-QuIC or Protein Misfolding Cyclic Amplification. Furthermore, comparison of the relative abilities of different prions to seed positive RT-QuIC reactions with bank vole and not other recombinant prion proteins allowed discrimination of prion strains such as classical and atypical L-type bovine spongiform encephalopathy, classical and atypical Nor98 scrapie in sheep, and sporadic and variant Creutzfeldt-Jakob disease in humans. Comparison of protease-resistant RT-QuIC conversion products also aided strain discrimination and suggested the existence of several distinct classes of prion templates among the many strains tested.

## Author Summary

Prion diseases are neurodegenerative disorders that propagate as multiple strains in a variety of mammalian species. The detection of all such prion types by a single ultrasensitive assay, such as the Real Time Quaking-induced Conversion (RT-QuIC) assay, would facilitate prion disease diagnosis, surveillance, and research. Here we show detection of minute amounts of 28 different prion types from humans, cattle, sheep, cervids and rodents, some of which were previously undetectable, using a single recombinant bank vole prion protein substrate. We also demonstrate the generation of prion type-dependent RT-QuIC conversion products which may help with prion strain discrimination and the characterization of distinct classes of prion templates. Finally, we describe a practical strategy for prion strain discrimination, e.g. classical and atypical L-type bovine spongiform encephalopathy; classical and atypical Nor98 sheep scrapie; and human sporadic and variant Creutzfeldt-Jakob disease. Thus, our study provides a basis for wide-ranging prion detection and strain discrimination.

## Introduction

Prion diseases, or transmissible spongiform encephalopathies, are neurodegenerative disorders that include Creutzfeldt-Jakob disease (CJD), Gerstmann-Straussler-Scheincker syndrome (GSS), fatal familial insomnia (FFI) and sporadic fatal insomnia (sFI) in humans, bovine spongiform encephalopathy (BSE) in cattle, scrapie in sheep, and chronic wasting disease (CWD) in cervids. The origin of prion diseases can be infectious, genetic or sporadic. Many prion diseases also have subtypes or strains that can be distinguished based on the *PRNP* (prion protein) genotype, transmission characteristics, clinical manifestations, neuropathological lesion profiles and/or biochemical properties of the disease-associated forms of prion protein (PrP^D^) [1–9]. PrP^D^, or a subset thereof, is the predominant molecular component of the infectious agent, or prion, which propagates itself by inducing misfolding of the hosts’ normal protease-sensitive prion protein, PrP^C^ or PrP^Sen^, into additional PrP^D^. This propagation mechanism appears to involve seeded, or templated, polymerization in which the given PrP^D^ conformation is imposed upon normally monomeric PrP^Sen^ molecules as they are recruited into growing PrP^D^ multimers [3,4].

PrP^D^ usually includes forms called PrP^Res^ that, unlike the normal PrP^Sen^, are partially resistant to digestion by proteinase K (PK). The banding pattern of PrP^Res^ in immunoblots can vary distinctively depending on the prion strain, host species and/or *PRNP* genotype. With most prion diseases the predominant 21-32-kDa variably glycosylated PrP^Res^ fragments observed on immunoblots extend from ragged N-termini between residues ~80–96 to the GPI-anchored C-terminus (e.g., at residue 231 in humans). In contrast, the PrP^Res^ associated with sheep Nor98 scrapie and human GSS linked to the P102L, F198S, A117V and H187R *PRNP* mutations include much smaller 6–14 kDa bands [10–12]. These bands are internal fragments with ragged N-and C-termini within residues ~80-~160 [13]. In cases of P102L GSS, brain tissue from some individuals can also give 21–32 kDa PrP^Res^ bands with the 7–8 kDa bands, while others give the 21–32 kDa PrP^Res^ bands but lack the 7–8 kDa bands. Hereafter, we will refer to the former cases as GSS P102L* and the latter as GSS P102L.

A major challenge for the prion disease field is the development of sufficiently practical and sensitive tests for routine prion disease detection and strain discrimination in medicine, agriculture, wildlife management and research. The Real Time Quaking Induced Conversion (RT-QuIC) assay, which is based on prion-seeded fibrillization of recombinant prion protein (rPrP^Sen^), is known to be highly specific and ultra-sensitive for detection of multiple human and animal prion diseases [14–20]. Moreover, like the amyloid seeding assay [21], RT-QuIC is more practical than comparably ultra-sensitive assays by being relatively rapid and based on a 96-well plate format with fluorescence readout [14,16]. Appropriate combinations of prion type and rPrP^Sen^ substrate have been important in the performance of various RT-QuIC assays [14,18–20,22–25]. For several types of prion disease, however, no effective rPrP^Sen^ substrate has been identified; these types include human GSS arising from P102L*, F198S, A117V and H187R *PRNP* mutations and the atypical sheep scrapie strain Nor98. Moreover, no single substrate has yet been shown to detect all of the different prion variants of humans, cattle, sheep, cervids and rodents.

One potential rPrP^Sen^ substrate that has not been described for RT-QuIC assays is based on the bank vole sequence. Bank voles [26], and transgenic mice that express bank vole (BV) PrP^Sen^ [27], are susceptible to an unusually wide range of prion strains from different species. Furthermore, PrP^Sen^ in bank vole brain tissue homogenates is a broadly reactive, but not universal, substrate for the highly sensitive protein misfolding cyclic amplification (PMCA) assay for prions [28]. Here we have tested the suitability of recombinant bank vole PrP^Sen^ (BV rPrP^Sen^), when expressed in *E*. *coli* and purified, as an RT-QuIC substrate. We have found so far that BV rPrP^Sen^ is a universally effective substrate for multiple prion strains from multiple species, and, most notably, for prions for which no effective substrate has been available. Furthermore, we have found that BV rPrP^Sen^-based RT-QuIC reactions give strain-dependent PK-resistant products in a manner that should further facilitate prion strain discrimination.

## Results

### Lack of detection of GSS and atypical scrapie subtypes associated with 6–14 kDa PrP^Res^ fragments using previously described rPrP^Sen^ constructs

**Fig 1:**
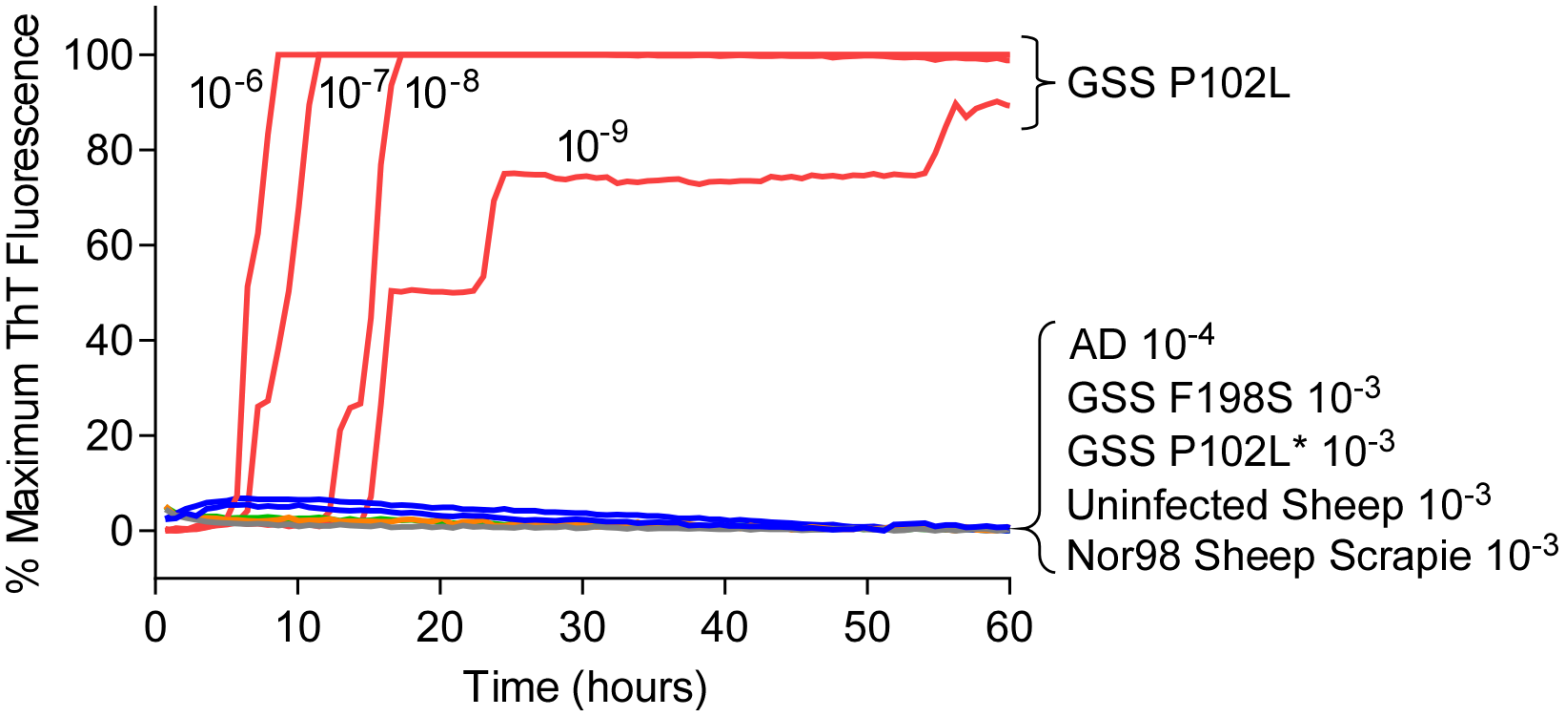
RT-QuIC detection of GSS P102L and lack of detection for GSS F198S, P102L* and sheep atypical Nor98 scrapie using hamster rPrP^Sen^ 90–231. Serial dilutions (10^-6^ to 10^-9^) of GSS P102L brain tissue dilutions were used to seed quadruplicate RT-QuIC reactions (red lines) with hamster 90–231 rPrP^Sen^ as the substrate, 300mM NaCl, 0.002% SDS. The same rPrP^Sen^ and RT-QuIC conditions listed above were used in reactions seeded with the designated brain tissue dilutions of GSS human patients with F198S (green line) or P102L* (gray line) *PRNP* point mutations, Alzheimer’s disease (AD, blue line), sheep without prion disease (blue line) or with Nor98 scrapie (orange line). Average ThT fluorescence readings from replicate wells for each type of sample were plotted as a function of time. Results are representative of similar findings from at least 10 independent experiments using hamster 90–231, hamster 23–231, human or hamster-sheep chimeric rPrP^Sen^ substrates.

Most mammalian PrP^Res^ types with predominant 21–32 kDa PrP^Res^ bands can seed Thioflavin T-positive (ThT) amyloid formation in RT-QuIC reactions [17,22,29–32] using at least one of the following substrates: Syrian golden hamster rPrP^Sen^ residues 90–231 [14,30,31], Syrian golden hamster rPrP^Sen^ 23–231 [18], human rPrP^Sen^ 23–231 [16], murine rPrP^Sen^ 23–231 [24] or hamster-sheep chimeric rPrP^Sen^ 23–231 [19,22]. For example, detection of human P102L GSS brain tissue using hamster rPrP^Sen^ 90–231 is shown in Fig 1. However, to date, no detection of RT-QuIC seeding activity has been reported using these rPrP^Sen^ substrates with cases of human GSS or sheep scrapie that give prominent low molecular weight PrP^Res^ fragments in immunoblots, despite extensive efforts. Specifically, these cases include human GSS with the F198S, A117V or H187R mutations and sheep Nor98 scrapie types giving 6–14 kDa PrP^Res^ fragments [10–13], and the human P102L-GSS with an ~8 kDa PrP^Res^ fragment (P102L*) [10]. Our inability to detect these prion types is exemplified in Fig 1 using 10^-3^ brain tissue dilutions of human GSS F198S and P102L* and sheep Nor98 scrapie with the hamster rPrP^Sen^ 90–231. In contrast, P102L GSS (without the ~8-kDa fragment) gave positive reactions with 1,000,000-fold smaller amounts of brain tissue.

### Detection of GSS F198S and A117V prion seeding activity using BV rPrP^Sen^

**Fig 2:**
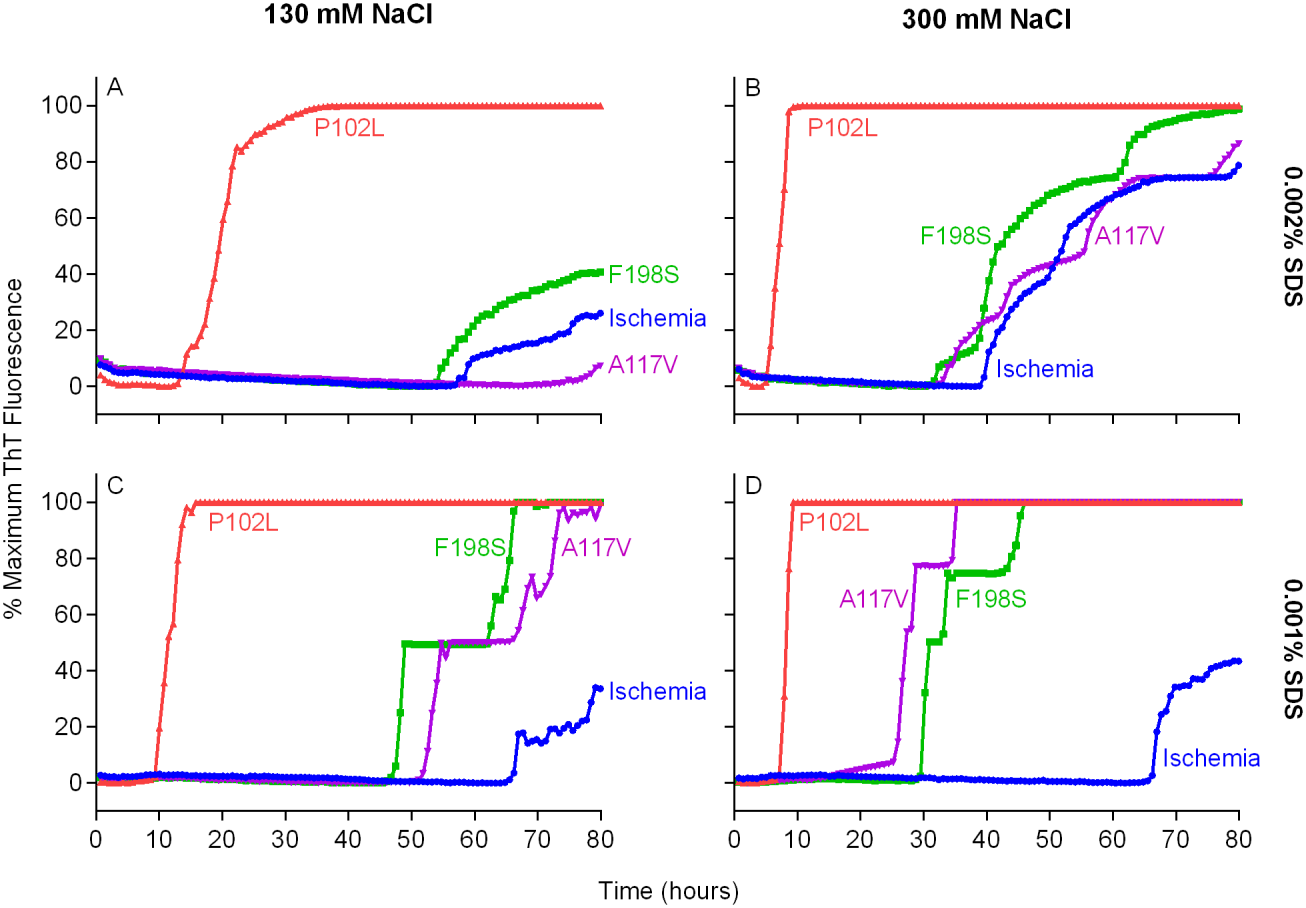
Detection of GSS P102L, F198S and A117V PrP^D^ types by RT-QuIC using BV rPrP^Sen^, 300mM NaCl and 0.001% SDS. Quadruplicate RT-QuIC reactions were seeded with 10^-4^ dilutions of human frontal cortex brain tissue from GSS patients with the P102L (red lines), F198S (green lines), or A117V (magenta lines) *PRNP* mutation. Negative control reaction were seeded with 10^-4^ dilutions of frontal cortex brain tissue from a cerebral ischemia patient (blue lines). A final SDS concentration of 0.002% (A and B) or 0.001% (C and D) in combination with 130 mM (A and C) or 300 mM (B and D) NaCl were used with BV rPrP^Sen^. Similar results were seen in three independent experiments. Traces from representative RT-QuIC experiments are the average of four replicate wells.

We then tested bank vole rPrP^Sen^ residues 23–230 (BV rPrP^Sen^) as a substrate to detect seeding activity of two human prion subtypes that have not been detectable previously by RT-QuIC, namely F198S- and A117V-GSS. Concurrently, we varied two parameters that we have shown to be influential, namely the concentrations of NaCl [14] and Sodium Dodecyl Sulfate (SDS) [30]. Each reaction was seeded with 10^-4^ dilutions of frontal cortex brain tissue from confirmed GSS cases carrying either the F198S or A117V mutation of the prion protein gene. We found that our standard concentrations of SDS (0.002%) in combination with either 130 or 300mM NaCl failed to allow a distinction in lag phase between prion positive and uninfected brain homogenate (BH) seeded reactions (Fig 2A and 2B). Lowering the SDS concentration to 0.001% with either 130 or 300mM NaCl improved this distinction between prion positive and uninfected BH seeded reactions (Fig 2C and 2D). However, using final concentrations of 300 mM NaCl and 0.001% SDS, provided much shorter lag phases in reactions seeded with the two GSS subtypes than with the cerebral ischemia negative control (Fig 2D). These results indicated that under these latter RT-QuIC conditions BV rPrP^Sen^ detected seeding activity associated with PrP^D^ conformers that had not otherwise been detectable by RT-QuIC or PMCA prion seed amplification techniques [33].

**Fig 3:**
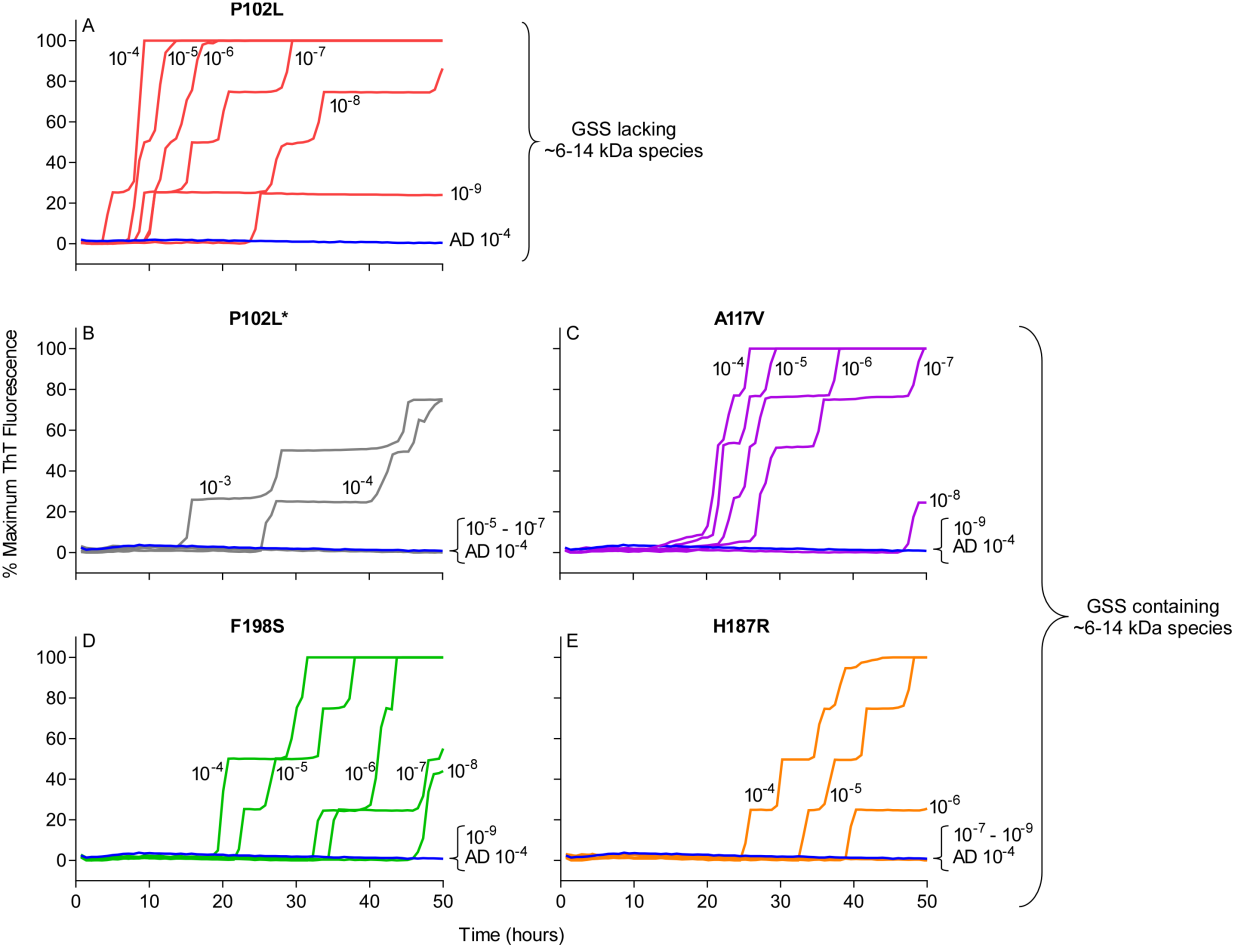
RT-QuIC sensitivity for detection of human GSS P102L, P102L*, A117V, F198S, and H187R seeding activity using BV rPrP^Sen^. The designated dilutions of frontal cortex brain tissue from the designated GSS P102L (A), P102L* (B), A117V (C), F198S (D), and H187R (E) patients were used to seed RT-QuIC reactions with 0.001% SDS and 300mM NaCl. Negative control reactions were seeded with Alzheimer’s disease (AD) brain tissue (A-E, blue lines). Representative data from one of three independent experiments is shown as the averages of fluorescence values from four replicate wells.

Next, we assessed the sensitivity of this new RT-QuIC assay for detecting GSS-associated prion seeding activity. Reactions were seeded with 10^-4^ to 10^-9^ dilutions of brain tissue from GSS patients carrying the P102L, P102L*, A117V, F198S and H187R mutation of the prion gene (Fig 3A–3E). A reaction time cutoff of 50h was chosen because in more than 20 independent RT-QuIC experiments seeded with negative control Alzheimer’s disease (AD) or cerebral ischemia brain homogenates, no positive RT-QuIC reactions were observed until after 55h (in rare wells). We detected GSS P102L, P102L*, A117V and F198S and H187R prion seeding activity in as little as 10^-9^, 10^-4^, 10^-8^, 10^-8^ and 10^-6^ dilutions of brain (frontal cortex) tissue dilutions, respectively (Fig 3).

### Detection of 28 different prion types/strains of human, sheep, cattle, deer, elk, mouse and hamster using BV rPrP^Sen^

**Fig 4:**
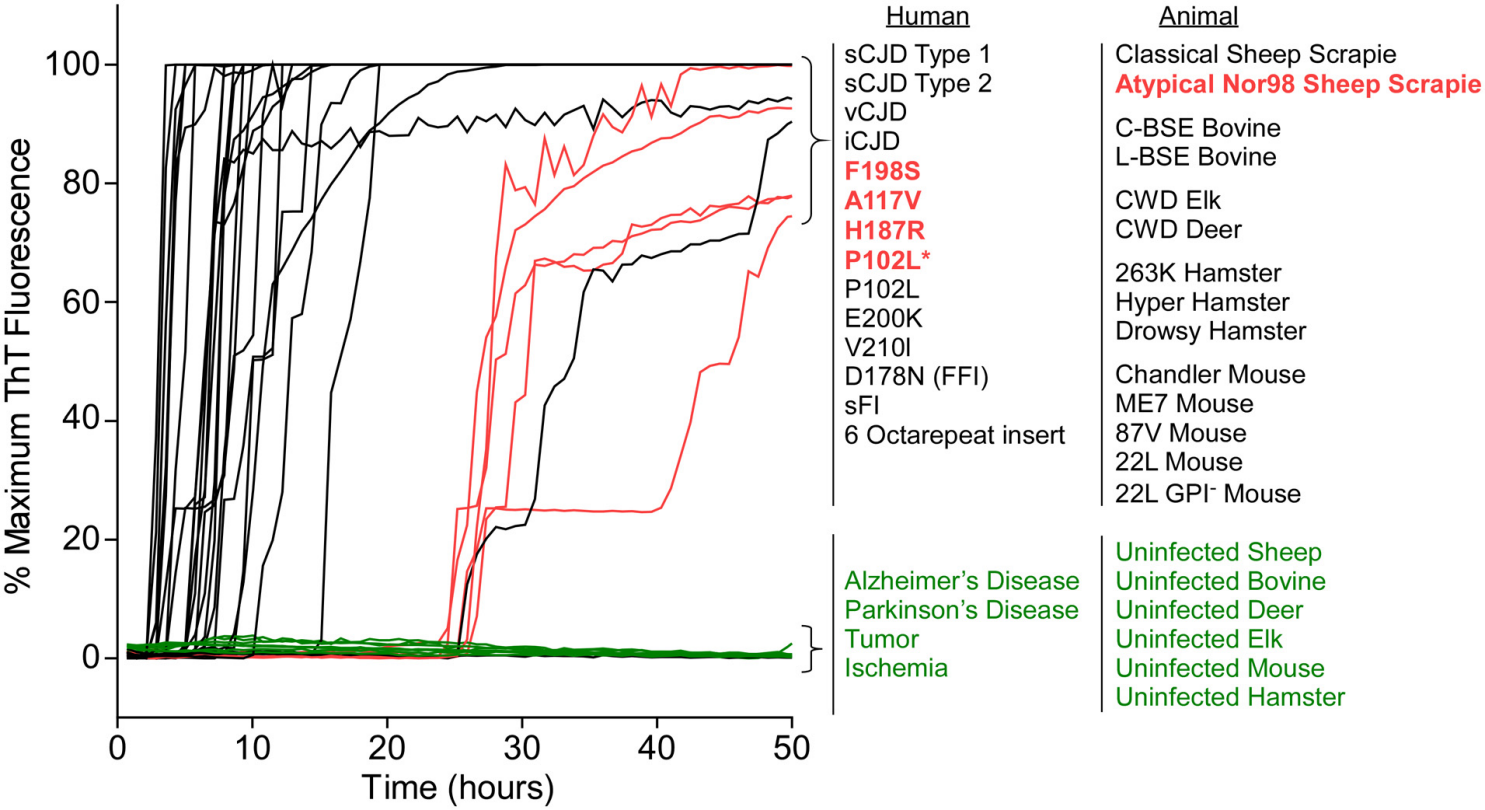
RT-QuIC detection of 28 types of prion seeds from 5 different species using new BV rPrP^Sen^ substrate. RT-QuIC reactions were seeded with 10^-4^ brain tissue dilutions of the indicated human and animal prion types in the presence of 300mM NaCl and 0.001% SDS. Equivalent dilutions of species- and brain region-matched samples from uninfected individuals were used as specificity controls (green). Prion types that have been detected previously by RT-QuIC using other substrate are indicated in black, whereas those that have only been detectable using our selected set of conditions and BV PrP^Sen^ are indicated in red. The traces show the average fluorescence from four replicate wells. Similar data were obtained from a minimum of three independent experiments with each prion type.

After finding that BV rPrP^Sen^ supported RT-QuIC detection of prion seeding activity from previously undetectable types of GSS, we tested whether BV rPrP^Sen^ could be used to detect other types of prion diseases. We tested 28 different types of prions in brain tissue from humans, sheep, mouse, hamster, cattle, elk, and deer (Tables 1 and 2) and found that all of them gave faster and stronger positive ThT fluorescence responses than a variety of uninfected negative control brain specimens (Fig 4). Among the 28 were the five prion types that have not been detectable by RT-QuIC under other conditions, namely human GSS F198S, A117V, H187R, and P102L* and sheep Nor98 scrapie (Fig 4, red traces). These results indicated that under these conditions, BV rPrP^Sen^ is the most broadly prion-seeded RT-QuIC substrate described to date.

**Table 1:** Human samples: diagnosis, *PRNP* genotype and brain regions.

Species	Diagnosis	Genotype	Brain Region		Detectable Dilution^+^
Human	Alzheimer's Disease	NA	Frontal Cortex		no reaction at 10^-4^
	Alzheimer's Disease	NA	Frontal Cortex		no reaction at 10^-4^
	Parkinson's Disease	NA	Frontal Cortex		no reaction at 10^-4^
	Cerebral Ischemia	NA	Frontal Cortex		no reaction at 10^-4^
	Tumor	NA	Frontal Cortex		no reaction at 10^-4^
	sCJD^1^ Type 1	129MM	Frontal Cortex		10^-8^
	sCJD Type 1	129VV	Frontal Cortex		
	sCJD Type 1	129MM	Frontal Cortex		
	sCJD Type 2	129MM	Frontal Cortex		
	sCJD Type 2	129VV	Frontal Cortex		
	sCJD Type 2	129MM	Frontal Cortex		
	vCJD^2^	NA	NA		10^-7^
	vCJD	NA	NA		
	vCJD	NA	NA		
	iCJD^3^ Type 1	129MM	Frontal Cortex	^‡^	
	iCJD Type 1	129MM	Frontal Cortex		
	iCJD Type 1	129MM	Frontal Cortex		
	iCJD Type 1	129MM	Frontal Cortex		
	iCJD Type 1	129MM	Frontal Cortex		
	iCJD Type 1	129MV	Frontal Cortex		
	iCJD Type 2	129MV	Frontal Cortex		
	iCJD Type 2	129VV	Frontal Cortex		
	E200K-gCJD^4^	129MM	Frontal Cortex		
	E200K-gCJD	129MM	Frontal Cortex		
	V210I-gCJD	129MM	Cerebellum	^#^	
	V210I-gCJD	129MM	Frontal Cortex		
	V210I-gCJD	129MM	Basal Ganglia		
	V210I-gCJD	129MM	Thalamus		
	Six Octarepeat Insert-gCJD	129VV	Cerebellum	^#^	
	Six Octarepeat Insert-gCJD	129VV	Frontal Cortex		
	Six Octarepeat Insert-gCJD	129VV	Basal Ganglia		
	Six Octarepeat Insert-gCJD	129VV	Thalamus		
	F198S GSS^5^	129MV	Frontal Cortex		10^-8^
	F198S-GSS	129VV	Frontal Cortex		
	F198S-GSS	129MV	Frontal Cortex		
	H187R-GSS	129MV	Frontal Cortex		10^-6^
	H187R-GSS	129MV	Frontal Cortex		
	A117V-GSS	129VV	Frontal Cortex		10^-8^
	A117V-GSS	129VV	Frontal Cortex		
	P102L*-GSS	129MV	Frontal Cortex		10^-4^
	P102L-GSS	129MV	Frontal Cortex		
	P102L-GSS	129MM	Frontal Cortex		10^-9^
	P102L-GSS	129MM	Frontal Cortex		
	D178N -FFI^6^	129MM	Frontal Cortex		
	D178N -FFI	129MM	Frontal Cortex		
	sFI^7^	129VV	Frontal Cortex		

Diagnosis, genotype and brain regions for human samples

^1^ Sporadic Creutzfeldt-Jakob disease

^**2**^ Variant Creutzfeldt-Jakob disease

^**3**^ Iatrogenic Creutzfeldt-Jakob disease

^**4**^ Genetic Creutzfeldt-Jakob disease

^**5**^ Gerstmann–Sträussler–Scheinker syndrome

^**6**^ Fatal familial insomnia

^**7**^ Sporadic fatal insomnia

^**‡**^ All French cases for which the source of prion contamination were growth hormone (7 patients) and dura matter graft (1 patient)

^**#**^Samples from the same patient

^+^Only dilutions of samples that were tested to end-point are displayed. Unless indicated all samples were tested at 10^-4^ tissue dilutions.

**Table 2:** Animal samples: prion disease, *Prnp* genotype and brain region.

Species	Prion Disease	Genotype	Brain Region	Detectable Dilution^+^
Rodent	Chandler Mouse	NA	Whole Brain	
	Chandler Mouse	NA	Whole Brain	
	22L Mouse	NA	Whole Brain	
	22L Mouse	NA	Whole Brain	
	22L GPI- Mouse	NA	Whole Brain	
	22L GPI- Mouse	NA	Whole Brain	
	ME7 Mouse	NA	Whole Brain	
	ME7 Mouse	NA	Whole Brain	
	87V Mouse	NA	Whole Brain	
	87V Mouse	NA	Whole Brain	
	Uninfected Mouse	NA	Whole Brain	no reaction at 10^-4^
	Uninfected Mouse	NA	Whole Brain	no reaction at 10^-4^
	Hyper Hamster	NA	Whole Brain	
	Hyper Hamster	NA	Whole Brain	
	Drowsy Hamster	NA	Whole Brain	
	Drowsy Hamster	NA	Whole Brain	
	263K Hamster	NA	Whole Brain	
	263K Hamster	NA	Whole Brain	
	Uninfected Hamster	NA	Whole Brain	no reaction at 10^-4^
	Uninfected Hamster	NA	Whole Brain	no reaction at 10^-4^
Bovine	L-BSE	NA	Brain Stem	
	L-BSE	NA	Brain Stem	
	C-BSE	NA	Frontal Cortex	
	C-BSE	NA	Frontal Cortex	
	Uninfected Bovine	NA	Brain Stem	no reaction at 10^-4^
	Uninfected Bovine	NA	Brain Stem	no reaction at 10^-4^
Cervid	CWD^1^ Elk	NA	Brain Stem	
	CWD Elk	NA	Brain Pool	
	Uninfected Elk	NA	Brain Stem	no reaction at 10^-4^
	CWD Deer	NA	Brain Pool	
	CWD Deer	NA	Cortex	
	CWD Deer	NA	Cortex	
	Uninfected Deer	NA	Brain Pool	no reaction at 10^-4^
	Uninfected Deer	NA	Cortex	no reaction at 10^-4^
Ovine	Classical Scrapie	VRQ/VRQ^†^	Cerebellum	10^-6^
	Classical Scrapie	ARQ/ARQ	Cerebellum	
	Classical Scrapie	ARQ/ARQ	Cerebellum	
	Classical Scrapie	ARQ/ARQ	Cerebellum	
	Classical Scrapie	ARQ/ARQ	Cerebellum	
	Classical Scrapie	ARQ/ARQ	Cerebellum	
	Classical Scrapie	VRQ/VRQ	Cerebellum	
	Classical Scrapie	ARQ/ARQ	Cerebellum	
	Nor98 Atypical Scrapie	ARR/AHQ^†^	Cerebellum	10^-7^
	Nor98 Atypical Scrapie	ARQ/ARQ	Cerebral Cortex	
	Nor98 Atypical Scrapie	ARQ/ARQ	Cerebellum	
	Nor98 Atypical Scrapie	ARQ/ARQ	Cerebellum	
	Nor98 Atypical Scrapie	ARQ/ARQ	Cerebellum	
	Nor98 Atypical Scrapie	ARQ/AHQ	Cerebellum	10^-3^
	Nor98 Atypical Scrapie	ARQ/AHQ	Cerebellum	
	Nor98 Atypical Scrapie	ARR/ARR	Cerebellum	
	Uninfected Sheep	ARQ/ARQ^†^	Cerebellum	no reaction at 10^-4^
	Uninfected Sheep	ARQ/ARQ	Cerebellum	no reaction at 10^-4^

Prion disease, *Prnp* genotype and brain region for animal samples.

^†^ Sheep *Prnp* genotype at codons 136/154/171

^+^Only dilutions of samples that were tested to end-point are displayed. Unless indicated all samples were tested at 10^-4^ brain tissue dilutions.

^1^Chronic Wasting Disease

### Prion strain/type-dependent RT-QuIC products from reactions using BV rPrP^Sen^

**Fig 5:**
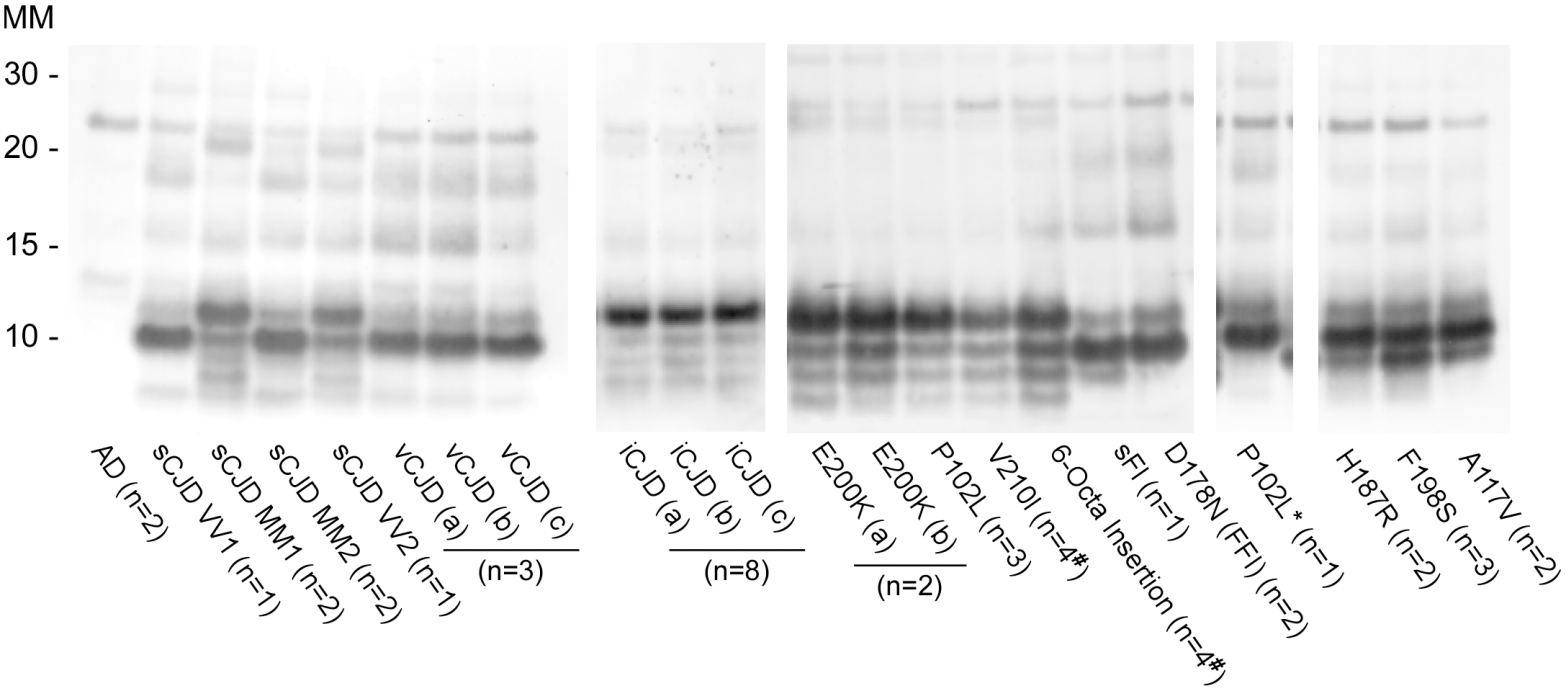
Western blot of BV rPrP^Res^ products from RT-QuIC reactions seeded with various human prion types. Reaction products were digested with 10μg/mL of PK. Immunoblots were probed with the C-terminal antibody R20 (hamster PrP epitope residues 218–231). Molecular mass (MM) is indicated in kilodaltons. Immunoblots are representative of one biological replicate (n = total biological replicates tested) each giving rPrP^Res^ banding profiles similar to that/those shown. ^(#)^ Samples for which cerebellum, frontal cortex, basal ganglia and thalamus from the same patient were analyzed. A minimum of two sets of RT-QuIC bank vole rPrP^Res^ products were generated from a given prion type and independently subjected to immunoblotting analysis.

Prion strain-dependence has not been observed previously in the immunoblot banding profile of PK-treated recombinant PrP^Res^ (rPrP^Res^) products of RT-QuIC reactions. However, using BV rPrP^Sen^ we observed consistently distinct products of RT-QuIC reactions seeded with different types of human prions (Fig 5 and Table 1). The observed banding patterns could be grouped based on the type of seed: GSS cases (F198S, A117V, H187R) with the ~8–14 Kda protease-resistant bands and sFI gave 2 bands: a major ~10kDa band and a ~6-9kDa band; the GSS (P102L), gCJD (E200K, V210I, six octarepeat insertion), and the iatrogenic CJD (iCJD) cases with ~21–32 kDa PrP^Res^ bands gave multiple bands with a major ~12 Kda band and multiple minor bands between ~6–10 kDa; variant CJD, GSS (P102L*) and FFI (D178N) cases gave a single predominant band at ~10 kDa; and sporadic CJD in some cases gave two bands between ~10–12 kDa, while in other cases gave a predominant band at ~ 10 kDa. Repeated analyses (>4) of individual sCJD cases indicated that they consistently seeded the formation of only one or the other of the latter two rPrP^Res^ products. This observation provided evidence that the different sCJD-seeded rPrP^Res^ products were dictated by differential templating activity in the tissue samples rather than stochastic events during the RT-QuIC reaction. Additionally, because these immunoblots used an antiserum to the C-terminus of PrP, the fragments likely differed primarily at their N-termini.

**Fig 6:**
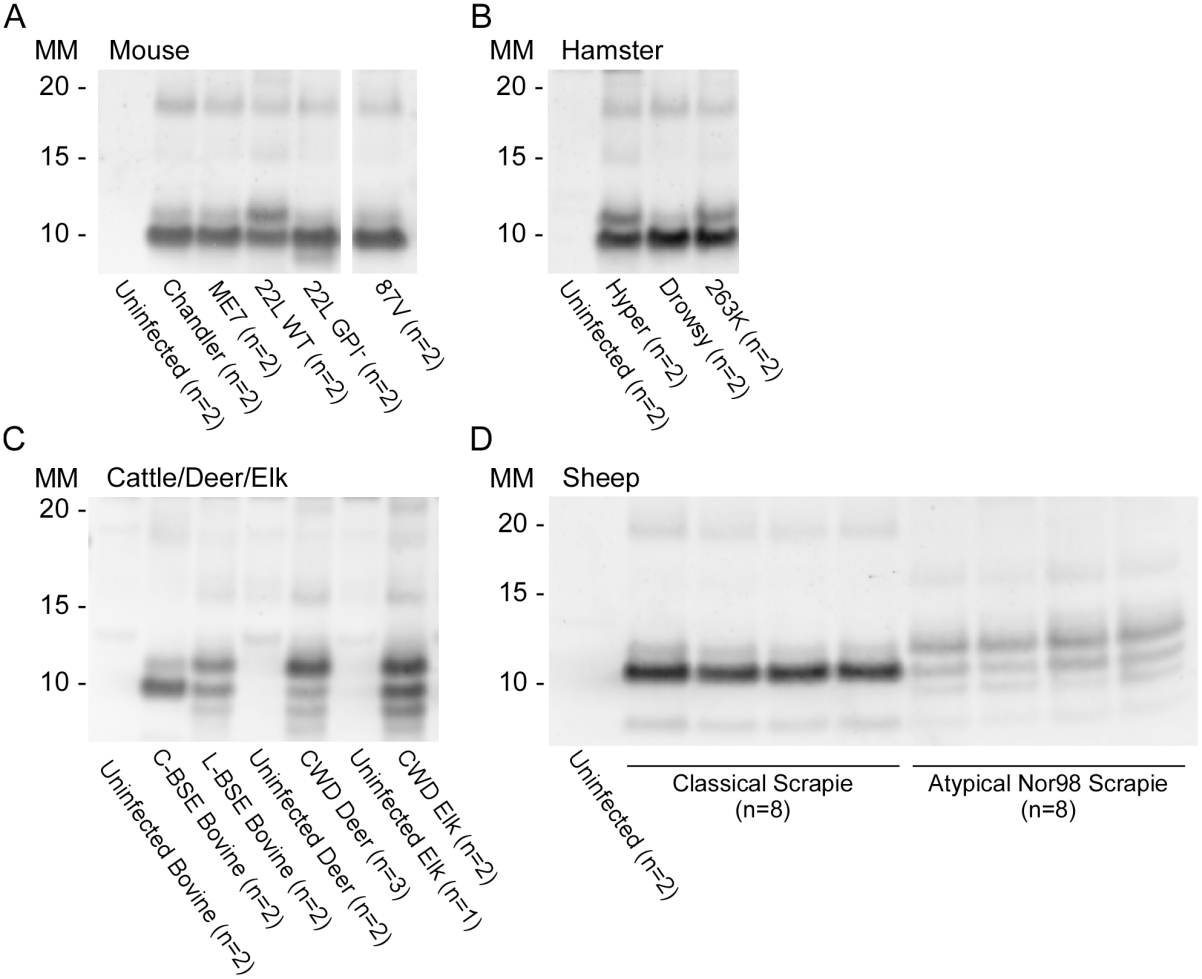
Western blot of BV rPrP^Res^ from RT-QuIC reactions seeded with rodent, bovine, cervine and sheep prion types. PK-treated RT-QuIC products from mouse (A), hamster (B), cattle (C), deer (C), elk (C) and sheep (D) prion seeds were probed with R20 (hamster PrP epitope residues 218–231). In (D), the classical scrapie-seeded reactions include those seeded with samples from *PRNP* VRQ/VRQ and ARQ/ARQ sheep (not designated). The Nor98-seeded reactions were seeded with samples from ARR/ARR, ARQ/AHQ and ARQ/ARQ sheep. RT-QuIC reactions and immunoblotting analysis for each of these types of prions were performed at least twice with similar results. The banding profiles shown are representative of multiple (n) independently tested biological replicates.

We further compared the BV rPrP^Res^ products of reactions seeded with different rodent, bovine, cervine and ovine prion strains (Table 2). As with the human prion seeds, we observed distinct strain-dependent BV rPrP^Res^ banding profiles from reactions seeded with different prion types. Mouse 22L scrapie-seeded BV rPrP^Res^ products consistently showed a ~10 and ~12 kDa PK-resistant band, whereas BV rPrP^Res^ products from reactions seeded with Chandler, ME7, 87V and anchorless 22L (22L GPI^-^) scrapie displayed a predominant ~10 kDa band (Fig 6A). The lack of the GPI anchor in the 22L GPI^-^ scrapie seed resulted in an RT-QuIC product that was distinct from the wild-type GPI-anchored 22L scrapie. Additionally, closely related hamster prion strains (Hyper and 263K; Fig 6B) showed similar BV rPrP^Res^ banding profiles (~10 and ~12 kDa PK-resistant bands) which were distinct from the Drowsy-seeded BV rPrP^Res^ products (primarily a ~10 kDa band; Fig 6B). Deer and elk CWD-seeded reactions each gave ~8, 9, 10, and 12 kDa bands, but differed in the relative intensities of the top two bands between the two (Fig 6C). Furthermore, distinct strain-dependent BV rPrP^Res^ banding profiles were observed between classical (C-BSE) and atypical (L-BSE) (~10 kDa vs. ~9, 10, and 12 kDa bands, respectively; Fig 6C), as well as between classical and atypical Nor98 sheep scrapie (~10 kDa vs. ~9, 10, and 12 kDa bands, respectively; Fig 6D). Collectively, these immunoblotting results suggested that certain human and animal prion diseases can be discriminated in part based on analysis of the rPrP^Res^ products of BV rPrP^Sen^-based RT-QuIC reactions.

### Detection and discrimination of classical and atypical BSE using BV and hamster rPrP^Sen^ substrates

**Fig 7:**
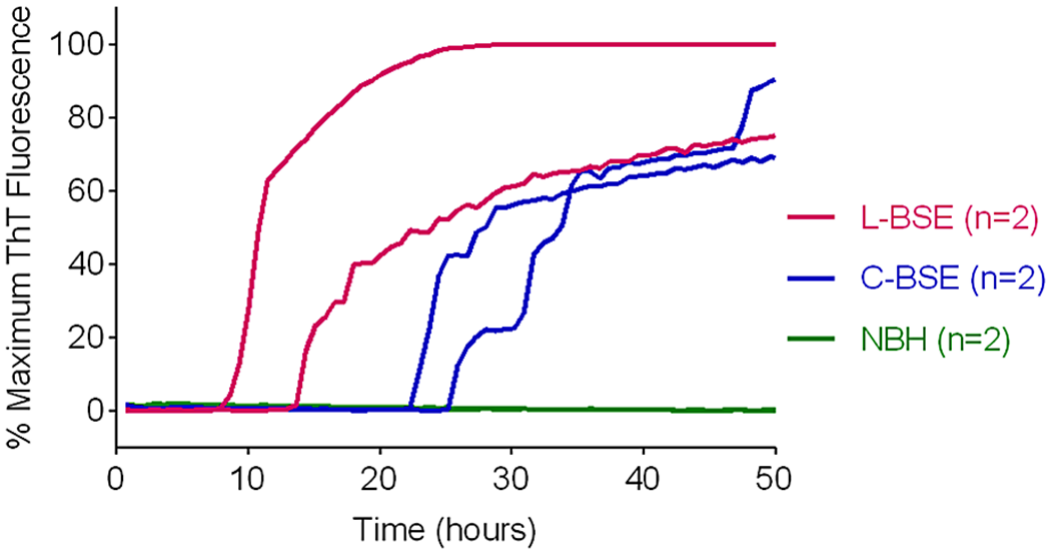
Detection of Classical (C-BSE) and atypical (L-type BSE) with BV rPrP^Sen^ substrate. RT-QuIC reactions were seeded with 10^-4^ brain tissue dilutions of brain stem (C-BSE, blue) or frontal cortex (L type-BSE, magenta) from Italian cattle. Negative control reactions (NBH, green) were seeded with 10^-4^ dilutions of frontal cortex or brain stem from uninfected cattle. BV rPrP^Sen^ was used as a substrate with 300mM NaCl and 0.001% SDS. RT-QuIC analysis was performed at least twice for each sample with similar results. Results are plotted as the averages from four replicate wells.

We previously reported that classical and atypical L-type BSE strains can be discriminated on the basis of relative RT-QuIC reactivities with hamster rPrP^Sen^ 90–231 and hamster-sheep chimeric rPrP^Sen^ 23–231 substrates [22]. Here we found that BV rPrP^Sen^ can similarly detect both classical and L-type BSE, providing an alternative substrate for discrimination between the two bovine strains. Specifically, detection of seeding activity with BV rPrP^Sen^ (Fig 7) but not with three other rPrP^Sen^ substrates that detected only L-type BSE, namely human 23–231, hamster 23–231 or hamster 90–231 [22], can be used to differentiate these two bovine prion types.

### Discrimination of classical and Nor98 sheep scrapie using BV and hamster-sheep chimeric rPrP^Sen^ substrates

**Fig 8:**
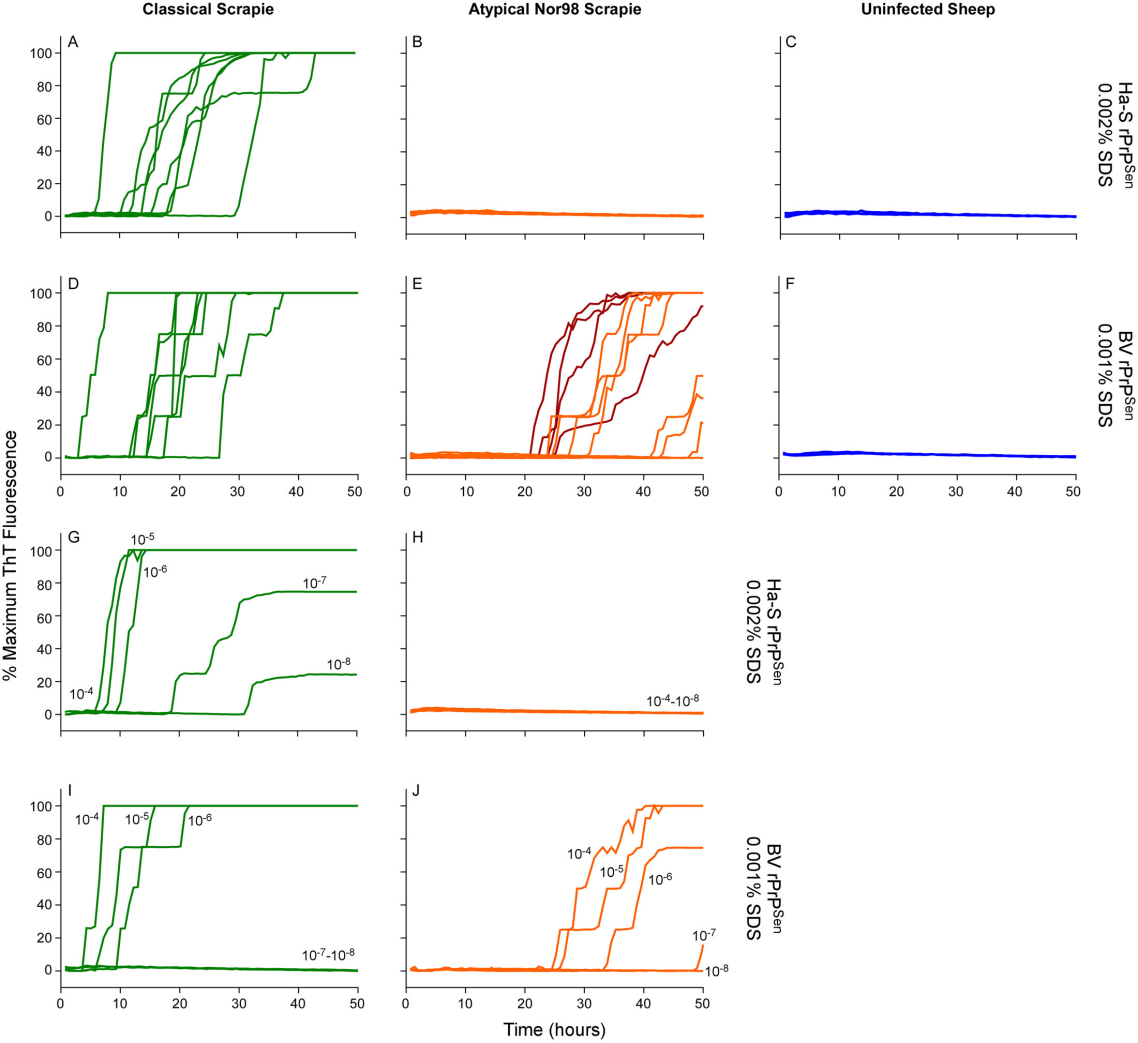
Detection, discrimination and sensitivities for detection of classical and Nor98 sheep scrapie with BV and Ha-S rPrP^Sen^ substrates. RT-QuIC reactions were seeded with dilutions of cerebellum or cerebral cortex from uninfected, classical or Nor98 atypical scrapie positive sheep. The Nor98 (ARR/AHQ, ARQ/ARQ, ARQ/AHQ and ARR/ARR *PRNP* genotypes) reactions were seeded with 10^-4^ (orange) brain tissue dilutions. Additional 10^-3^ (red) brain tissue dilutions are also shown for weaker samples. Classical sheep scrapie brain tissue from eight animals (ARQ/ARQ, VRQ/VRQ *PRNP* genotypes) was diluted 10^-4^ (green, A and D). Equivalent dilutions of cerebellum or frontal cortex brain tissue dilutions (ARQ/ARQ *PRNP* genotypes) were used as specificity controls (blue, C and F). Either Ha-S rPrP^Sen^ (300mM NaCl and 0.002% SDS; A-C) or BV rPrP^Sen^ (300mM NaCl and 0.001% SDS; D-F) were used as substrates. Brain homogenates from classical scrapie positive sheep (green, VRQ/VRQ) and atypical Nor98 scrapie positive sheep (orange, ARR/AHQ) were serially diluted (10^-4^ to 10^-8^) for RT-QuIC analysis using Ha-S rPrP^Sen^ with 300mM NaCl and 0.002% SDS (G and H) or BV rPrP^Sen^ with 300mM NaCl and 0.001% SDS (I and J) substrates. RT-QuIC testing was performed independently twice with similar results. Traces show averages of quadruplicate wells.

Having detected Nor98 sheep scrapie with BV rPrP^Sen^, (Fig 4) we tested whether a strategy similar to the one described above for C- vs. L-type BSE using different rPrP^Sen^ substrates would allow discrimination of Nor98 and classical sheep scrapie. Brain tissue from eight sheep with classical scrapie [ARQ/ARQ (n = 6), VRQ/VRQ (n = 2) PrP genotypes, Table 2] were readily detected using the hamster-sheep chimeric rPrP^Sen^ 23–231 within ~40hs (Fig 8A). However, brain tissue from eight cases of Nor98 scrapie [ARR/AHQ (n = 1), ARQ/ARQ (n = 4), ARQ/AHQ (n = 2) and ARR/ARR (n = 1) genotypes, Table 2] gave no positive responses using the same substrate (Fig 8B). In contrast, consistent with the results in Fig 4, seven of these cases gave positive responses when BV rPrP^Sen^ was used in reactions seeded with 10^-4^ brain tissue dilutions (Fig 8A–8E, orange lines) and those that were weaker or not detected were positive when seeded with 10^-3^ dilutions (Fig 8A–8E, red lines). To compare the sensitivities of the assay for detection of classical and atypical scrapie using these two substrates, we diluted representative brain homogenates from classical and Nor98 scrapie positive sheep (Fig 8G–8J) and tested them using both BV and Ha-S rPrP^Sen^. We detected classical scrapie down to 10^-8^ dilutions using Ha-S rPrP^Sen^ and down to 10^-6^ using BV rPrP^Sen^. Consistent with the data in Fig 8, no fluorescence increases were seen in reactions seeded with the same dilutions of a Nor98 atypical scrapie sample when using Ha-S rPrP^Sen^. In contrast, parallel reactions with BV rPrP^Sen^ gave positive reactions when seeded with Nor98 brain dilutions down to 10^-6^–10^-7^, indicating that BV rPrP^Sen^ is ~1,000-fold more sensitive at detecting Nor98 scrapie than is Ha-S rPrP^Sen^. Collectively, these results suggest that if an ovine brain sample gives a positive RT-QuIC response with BV rPrP^Sen^, it should give a stronger positive reaction with Ha-S rPrP^Sen^ if it contains classical scrapie, but a negative, or at least much weaker, reaction if it contains Nor98 scrapie.

### Detection and discrimination of human sCJD and vCJD using BV and hamster 23–231 rPrP^Sen^ substrates

**Fig 9:**
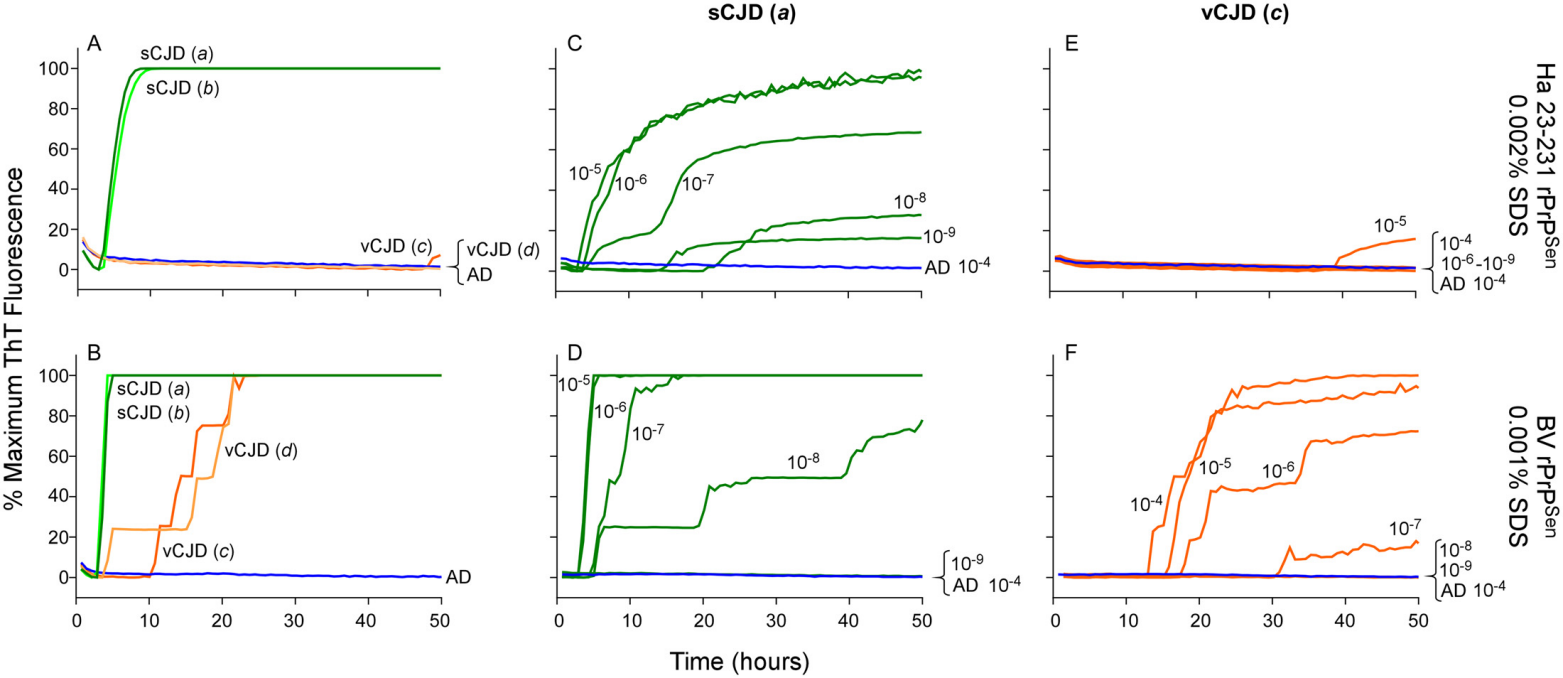
Detection, discrimination and sensitivities for detection of human sCJD and vCJD with hamster 23–231 and BV rPrP^Sen^ substrates. Dilutions (10^-4^) of frontal cortex brain tissue from two confirmed sCJD (Case *a* and *b*, green) and two vCJD cases (Case *c* and *d*, orange) were used to seed RT-QuIC reactions. Testing was performed using either hamster (23–231) in the presence of 300mM NaCl and 0.002% SDS (A) or BV PrP^Sen^ in the presence of 300mM NaCl and 0.001%SDS (B). Brain homogenates from one sCJD (Case *a*) and one vCJD (Case *c*) patient were serially diluted (10^-5^ to 10^-8^) for RT-QuIC analysis. Hamster 23–231 (C and E) and BV rPrP^Sen^ (D and F) were used as a substrate. These samples were tested in three independent experiments with similar results. Traces represent the averages of four replicate wells.

To investigate the discrimination of two non-genetic human prion strains, we tested 10^-4^ brain tissue dilutions from two confirmed cases of Type 1 sCJD (Fig 9A and 9B, Cases *a* and *b*, green lines) and two cases of vCJD (Fig 9A and 9B, Cases *c* and *d*, orange lines). We used previously described SDS conditions (0.002% final concentration of SDS; [23]) with hamster 23–231 rPrP^Sen^, and 0.001% SDS with BV rPrP^Sen^, both in the presence of 300mM NaCl. We observed rapid amplification of prion seeding activity in the two Type 1 sCJD samples when using either hamster 23–231 or BV rPrP^Sen^ (Fig 9A and 9B). Our detection of the sCJD samples with the hamster 23–231 substrate was consistent with previous demonstrations that all sCJD subtypes are detectable with this substrate [23,30,34]. No increase in ThT fluorescence was seen in vCJD-seeded hamster 23–231 rPrP^Sen^ RT-QuIC reactions (Fig 9A). However, in accordance with the results shown in Fig 4, seeding activity was detected in both vCJD samples using BV rPrP^Sen^ (Fig 9B). Thus, sporadic and variant CJD sample were discriminated by differential reactivities with the BV and hamster 23–231 rPrP^Sen^ substrates.

Next we compared the RT-QuIC sensitivities for detection of sCJD and vCJD brain homogenates using hamster and BV rPrP^Sen^. We performed end-point dilution RT-QuIC analysis of brain tissue from sCJD (Case *a*) and vCJD (Case *c*) (Fig 9C–9F, green and orange lines, respectively). We detected sCJD down to 10^-8^/10^-9^ with hamster 23–231 rPrP^Sen^, (Fig 9C) and 10^-8^ with BV rPrP^Sen^ (Fig 9D). Although sCJD gave slightly slower amplification kinetics with hamster 23–231 rPrP^Sen^ (Fig 9C) compared to BV rPrP^Sen^ (Fig 9D), the overall sensitivities using the two substrates were comparable. In contrast, markedly different sensitivities were observed with the two substrates in the vCJD-seeded reactions. Specifically, only weak seeding activity was occasionally detected in 10^-4^ or 10^-5^ brain dilutions with hamster 23–231 rPrP^Sen^ (representative data in Fig 9E), but fast and sensitive detection of vCJD seeding activity down to 10^-7^ brain tissue dilution was observed using BV rPrP^Sen^ (Fig 9F). These results suggest that BV rPrP^Sen^ is 100–10,000-fold more sensitive than hamster 23–231 rPrP^Sen^ in detecting vCJD brain derived prion seeding activity. Collectively, these findings further support the potential broad applicability of a BV rPrP^Sen^ prion discrimination strategy to a variety of prion types.

## Discussion

The lack of practical and cost-effective tests that are sensitive enough to detect the lowest infectious levels of prions has long been a major impediment in coping with prion diseases. Rapid commercially available immunoassays have allowed post-mortem detection of prion infections in high-titered tissues such as brain or lymphoid tissues, but diagnostic specimens that are most readily accessible in living hosts, such as blood, CSF and nasal brushings, have much lower prion titers that are undetectable with these assays. In contrast, RT-QuIC assays have been highly effective in detecting prion seeding activity in such low-titered specimens, and are being widely implemented as state-of-the-art diagnostic tests for humans and animals [16,18–20,25,34–37]. Moreover, recent improvements have increased the speed and sensitivity of RT-QuIC assays such that sCJD testing based on human CSF samples can now be performed in a matter of hours rather than days [30].

In our experience, the most demanding and costly requirement for RT-QuIC testing is the availability of suitable rPrP^Sen^ substrates. Prior to the present study, testing facilities would typically have to produce or procure multiple rPrP^Sen^ sequences to be able to test for multiple prion types. However, we have now shown that all of the prion diseases that we have tested so far from humans and other mammals can be detected sensitively by using BV rPrP^Sen^ (Fig 4). This provides a useful platform for broad-based prion detection and strain discrimination. Thus, we envision that most initial screening for the presence of a wide variety of prions could be performed using BV rPrP^Sen^. Once a prion-infected sample from a given host species is identified, one could then often discriminate between strains by targeted use of another rPrP^Sen^ substrate that is known to be differentially sensitive to seeding by prion strains of that host species (Figs 7–9) and/or by performing immunoblots of the PK-resistant RT-QuIC products of the reactions (Figs 5 and 6).

Although we have demonstrated detection of a wide variety of prion types, the relative sensitivities of BV rPrP^Sen^-based RT-QuIC for brain homogenates of hosts with different prion diseases is presumably dependent on the concentrations of PrP^D^ in the tissue samples. Clearly PrP^D^ concentrations may vary markedly between individuals and different regions of the brain as a function of strain. Furthermore, because PrP^D^ can vary markedly in its properties, e.g. amyloid vs. non-amyloid, protease-sensitive vs. resistant, small vs. large particles, infectious vs. non-infectious, it is probable that the RT-QuIC seeding activity will vary per unit PrP^D^ between different prion strains and tissue sources. Thus, although we have shown the potential for BV rPrP^Sen^-based RT-QuIC to detect and help discriminate prion strains, much additional work with each type of prion and sample type will be required to better establish the quantitative relationships between RT-QuIC seeding activity and the levels of various types of PrP^D^ in different tissues of diagnostic or scientific interest.

Since the inception of prion-seeded cell-free PrP conversion reactions [38], striking sequence- and strain-specificities have been observed that appeared to correlate, at least largely, with transmission barriers and strain phenotypes of prion diseases *in vivo* [3,39–41]. Indeed, sequence differences of as little as a single residue between the PrP^D^ seed and PrP^Sen^ substrate can block PrP^Res^ formation in such cell-free reactions [42], as it can in scrapie-infected cells [43] and *in vivo* [44]. However, RT-QuIC assays have tended to be less constrained by such sequence differences [14]. We reason that this is due in part to the fact that in RT-QuIC reactions, it is only the C-terminal residues ~160–231 of the substrate molecules that must refold into the PK-resistant amyloid core [45] to give a positive reaction, i.e., an increase in ThT fluorescence. In contrast, earlier cell-free conversion [38,46,47] and PMCA reactions [48] have used the immunoblot-based detection of much larger PK-resistant cores, typically comprised of residues ~90–231, as a positive readout. Thus, much more extensive packing of more N-proximal residues is required in the latter reactions, as it is *in vivo*, giving more opportunities for sequence differences between seed and substrate to influence conversion. Nonetheless, despite the lower sequence specificity of RT-QuIC reactions, we and others have observed multiple examples of rPrP^Sen^ substrates that can be converted by some types of prion seeds and not others [22,23]. Therefore, we were surprised to find that BV rPrP^Sen^ can be induced to convert to ThT-positive amyloid by every type of prion-associated seed that we have tried so far (n = 28), including several that had never before been detected by RT-QuIC or PMCA. We also did not anticipate that different PK-resistant BV rPrP^Res^ products of RT-QuIC reactions would be seeded with different prion strains from a single host species, because we had never seen such distinct templating with the many other rPrP^Sen^ substrates that we have tested. These findings suggest that BV rPrP^Sen^-based RT-QuIC reactions may provide a new means of probing the strain-dependent heterogeneity of prion seeding activities and conformational templates. However, overall, the RT-QuIC technology has been established largely for the practical purposes of rapid, sensitive prion disease-associated seed detection rather than the *in vitro* recapitulation of prion propagation. As such, the RT-QuIC tests have not been developed to reflect prion transmission barriers or strain-specificities. In any case, the availability of BV rPrP^Sen^ as an apparently universal RT-QuIC substrate may markedly improve the practicality, efficiency and cost-effectiveness of detecting and discriminating prions.

## Materials and Methods

### Ethics statement

Brain tissue from scrapie-infected mice and hamsters (Table 2) were collected under Protocols 2013–030 and 2010–045, respectively, that were approved by the Rocky Mountain Laboratories Animal Care and Use Committee. Human brain tissues (Table 1) were obtained from the National Prion Disease Pathology Surveillance Center (USA). Brain tissue from humans with vCJD (Table 1) was obtained from the National Institute for Biological Standards and Controls (UK) repository. No human samples were collected expressly for this study, but were instead obtained from the existing collections noted above with approval, as needed, under exemption #1197 from the NIH Office of Human Subjects Research. All human samples were, and remain, anonymized to the investigators at Rocky Mountain Laboratories where the RT-QuIC testing was performed.

### Protein expression and purification

Recombinant prion protein (rPrP^Sen^) substrates were purified as previously described [49]. Briefly, PrP DNA sequences encoding for Syrian golden hamster (residues 23 to 231; accession no. K02234; or residues 90–231), Bank Vole (residues 23 to 230; Methionine at residue 109; accession no. AF367624) or hamster-sheep chimera (Syrian hamster residues 23 to 137 followed by sheep residues 141 to 234 of the R_154_Q_171_ polymorph [accession no. AY907689]) prion protein genes were ligated into the pET41 vector (EMD Biosciences). Vectors were transformed into Rosetta (DE3) *Escherichia coli* and were grown in Luria broth medium in the presence of kanamycin and chloramphenicol. Protein expression was induced using the autoinduction system [50,51] and was purified from inclusion bodies under denaturing conditions using Ni-nitrilotriacetic acid (NTA) superflow resin (Qiagen) with an ÄKTA fast protein liquid chromatographer (GE Healthcare Life Sciences). The protein was refolded on the column using a guanidine HCl reduction gradient and eluted using an imidazole gradient as described [49]. The eluted protein was extensively dialyzed into 10 mM sodium phosphate buffer (pH 5.8), filtered (0.22-μm syringe filter [Fisher]) and stored at -80°C. Protein concentration was determined by measuring absorbance at 280 nm.

### Brain homogenate preparations

Brain homogenates (BH; 10% w/v, Tables 1 and 2) were prepared as previously described [14] and stored at -80°C. For RT-QuIC analysis BHs were serially diluted in 0.1% SDS (sodium dodecyl sulfate, Sigma)/N2 (Gibco)/PBS as previously reported (25), or where indicated the last dilutions were performed to a final concentration of 0.05% SDS/N2/PBS.

### RT-QuIC protocol

RT-QuIC reactions were performed as previously described [14]. Reaction mix was composed of 10 mM phosphate buffer (pH 7.4), 300 or 130 mM NaCl, 0.1 mg/ml rPrP^Sen^, 10 μM thioflavin T (ThT), 1 mM ethylenediaminetetraacetic acid tetrasodium salt (EDTA), and 0.002% or 0.001% SDS. NaCl and SDS concentrations were varied where indicated. Aliquots of the reaction mix (98 μL) were loaded into each well of a black 96-well plate with a clear bottom (Nunc) and seeded with 2 μL of indicated BH dilutions. The plate was then sealed with a plate sealer film (Nalgene Nunc International) and incubated at 42°C in a BMG FLUOstar Omega plate reader with cycles of 1 min shaking (700 rpm double orbital) and 1 min rest throughout the indicated incubation time. ThT fluorescence measurements (450 +/-10 nm excitation and 480 +/-10 nm emission; bottom read) were taken every 45 min.

To compensate for minor differences in baselines between fluorescent plate readers and across multiple experiments, data sets were normalized to a percentage of the maximal fluorescence response (260,000 rfu) of the plate readers after subtraction of the baseline, as described [34], and plotted versus reaction time. Reactions were classified as RT-QuIC positive base on criteria similar to those previously described for RT-QuIC analyses of brain specimens [14,34].

### Proteinase K (PK) digestion of RT-QuIC products and immunoblotting

RT-QuIC reaction products were collected from the plates by extensive scraping and pipetting and treated with 10 μg/ml Proteinase K (PK) for 1 hour at 37°C with 400 rpm orbital shaking. Equal volumes of PK-treated reactions were run on 12% Bis-Tris NuPAGE gels (Invitrogen). Proteins were transferred to an Immobilon P membrane (Millipore) using the iBlot Gel Transfer System (Invitrogen). Membranes were probed with R20 primary antiserum (hamster epitope: residues 218–231) [52] diluted 1:15,000 and visualized with the Attophos AP fluorescent substrate system (Promega) according to the manufacturer's recommendations.

## References

[1] Bruce M, Dickinson AG (1979) Biological stability of different classes of scrapie agent In: Prusiner SB, Hadlow WJ, editors. Slow transmissible diseases of the nervous system. New York: Academic Press pp. 71–86.

[2] Bessen RA, Marsh RF (1994) Distinct PrP properties suggest the molecular basis of strain variation in transmissible mink encephalopathy. JVirol 68: 7859–7868. 7966576 10.1128/jvi.68.12.7859-7868.1994 PMC237248

[3] Bessen RA, Kocisko DA, Raymond GJ, Nandan S, Lansbury PT Jr., et al (1995) Nongenetic propagation of strain-specific phenotypes of scrapie prion protein. Nature 375: 698–700. 7791905 10.1038/375698a0

[4] Telling GC, Parchi P, DeArmond SJ, Cortelli P, Montagna P, et al (1996) Evidence for the conformation of the pathologic isoform of the prion protein enciphering and propagating prion diversity. Science 274: 2079–2082. 8953038 10.1126/science.274.5295.2079

[5] Caughey B, Raymond GJ, Bessen RA (1998) Strain-dependent differences in beta-sheet conformations of abnormal prion protein. JBiolChem 273: 32230–32235. 9822701 10.1074/jbc.273.48.32230

[6] Safar J, Wille H, Itri V, Groth D, Serban H, et al (1998) Eight prion strains have PrP(Sc) molecules with different conformations [see comments]. NatMed 4: 1157–1165. 9771749 10.1038/2654

[7] Parchi P, Giese A, Capellari S, Brown P, Schulz-Schaeffer W, et al (1999) Classification of sporadic Creutzfeldt-Jakob disease based on molecular and phenotypic analysis of 300 subjects. AnnNeurol 46: 224–233. 10443888

[8] Parchi P, Zou W, Wang W, Brown P, Capellari S, et al (2000) Genetic influence on the structural variations of the abnormal prion protein. ProcNatlAcadSciUSA 97: 10168–10172. 10963679 10.1073/pnas.97.18.10168 PMC27779

[9] Collinge J, Clarke AR (2007) A general model of prion strains and their pathogenicity. Science 318: 930–936. 17991853 10.1126/science.1138718

[10] Monaco S, Fiorini M, Farinazzo A, Ferrari S, Gelati M, et al (2012) Allelic origin of protease-sensitive and protease-resistant prion protein isoforms in Gerstmann-Straussler-Scheinker disease with the P102L mutation. PLoS One 7: e32382 10.1371/journal.pone.0032382 22384235 PMC3285667

[11] Piccardo P, Liepnieks JJ, William A, Dlouhy SR, Farlow MR, et al (2001) Prion proteins with different conformations accumulate in Gerstmann-Straussler-Scheinker disease caused by A117V and F198S mutations. AmJPathol 158: 2201–2207. 11395398 10.1016/S0002-9440(10)64692-5 PMC1891977

[12] Gotte DR, Benestad SL, Laude H, Zurbriggen A, Oevermann A, et al (2011) Atypical scrapie isolates involve a uniform prion species with a complex molecular signature. PLoS One 6: e27510 10.1371/journal.pone.0027510 22096587 PMC3214077

[13] Pirisinu L, Nonno R, Esposito E, Benestad SL, Gambetti P, et al (2013) Small ruminant nor98 prions share biochemical features with human gerstmann-straussler-scheinker disease and variably protease-sensitive prionopathy. PLoS One 8: e66405 10.1371/journal.pone.0066405 23826096 PMC3691246

[14] Wilham JM, Orrú CD, Bessen RA, Atarashi R, Sano K, et al (2010) Rapid End-Point Quantitation of Prion Seeding Activity with Sensitivity Comparable to Bioassays. PLoS Pathogens 6: e1001217 10.1371/journal.ppat.1001217 21152012 PMC2996325

[15] Atarashi R, Sano K, Satoh K, Nishida N (2011) Real-time quaking-induced conversion: A highly sensitive assay for prion detection. Prion 5: 150–153. 10.4161/pri.5.3.16893 21778820 PMC3226039

[16] Atarashi R, Satoh K, Sano K, Fuse T, Yamaguchi N, et al (2011) Ultrasensitive human prion detection in cerebrospinal fluid by real-time quaking-induced conversion. Nature Medicine 17: 175–178. 10.1038/nm.2294 21278748

[17] Orru CD, Wilham JM, Vascellari S, Hughson AG, Caughey B (2012) New generation QuIC assays for prion seeding activity. Prion 6: 147–152. 10.4161/pri.19430 22421206 PMC7082091

[18] McGuire LI, Peden AH, Orru CD, Wilham JM, Appleford NE, et al (2012) RT-QuIC analysis of cerebrospinal fluid in sporadic Creutzfeldt-Jakob disease. Annals of Neurology 72: 278–285. 10.1002/ana.23589 22926858 PMC3458796

[19] Cramm M, Schmitz M, Karch A, Zafar S, Varges D, et al (2015) Characteristic CSF prion seeding efficiency in humans with prion diseases. Mol Neurobiol 51: 396–405. 10.1007/s12035-014-8709-6 24809690 PMC4309904

[20] Cramm M, Schmitz M, Karch A, Mitrova E, Kuhn F, et al (2015) Stability and Reproducibility Underscore Utility of RT-QuIC for Diagnosis of Creutzfeldt-Jakob Disease. Mol Neurobiol. 10.1007/s12035-015-9133-2 PMC4789202 25823511

[21] Colby DW, Zhang Q, Wang S, Groth D, Legname G, et al (2007) Prion detection by an amyloid seeding assay. Proc Natl Acad Sci USA 104: 20914–20919. 18096717 10.1073/pnas.0710152105 PMC2409241

[22] Orru CD, Favole A, Corona C, Mazza M, Manca M, et al (2015) Detection and Discrimination of Classical and Atypical L-type Bovine Spongiform Encephalopathy by Real-Time Quaking-Induced Conversion. J Clin Microbiol. 10.1128/JCM.02906-14 PMC4365258 25609728

[23] Peden AH, McGuire LI, Appleford NE, Mallinson G, Wilham JM, et al (2012) Sensitive and specific detection of sporadic Creutzfeldt-Jakob disease brain prion protein using real-time quaking induced conversion. JGenVirol 93: 438–449. 10.1099/vir.0.033365-0 PMC3352348 22031526

[24] Vascellari S, Orru CD, Hughson AG, King D, Barron R, et al (2012) Prion seeding activities of mouse scrapie strains with divergent PrPSc protease sensitivities and amyloid plaque content using RT-QuIC and eQuIC. PLoS ONE 7: e48969 10.1371/journal.pone.0048969 23139828 PMC3489776

[25] Sano K, Satoh K, Atarashi R, Takashima H, Iwasaki Y, et al (2013) Early detection of abnormal prion protein in genetic human prion diseases now possible using real-time QUIC assay. PLoSONE 8: e54915 10.1371/journal.pone.0054915 23372790 PMC3556051

[26] Nonno R, Di Bari MA, Cardone F, Vaccari G, Fazzi P, et al (2006) Efficient transmission and characterization of Creutzfeldt-Jakob disease strains in bank voles. PLoS Pathog 2: e12 16518470 10.1371/journal.ppat.0020012 PMC1383487

[27] Watts JC, Giles K, Patel S, Oehler A, DeArmond SJ, et al (2014) Evidence that bank vole PrP is a universal acceptor for prions. PLoS Pathog 10: e1003990 10.1371/journal.ppat.1003990 24699458 PMC3974871

[28] Cosseddu GM, Nonno R, Vaccari G, Bucalossi C, Fernandez-Borges N, et al (2011) Ultra-efficient PrP(Sc) amplification highlights potentialities and pitfalls of PMCA technology. PLoSPathog 7: e1002370. 10.1371/journal.ppat.1002370 PMC3219717 22114554

[29] Orru CD, Caughey B (2011) Prion seeded conversion and amplification assays. TopCurrChem 305: 121–133. 10.1007/128_2011_184 PMC6319375 21678135

[30] Orru CD, Groveman BR, Hughson AG, Zanusso G, Coulthart MB, et al (2015) Rapid and Sensitive RT-QuIC Detection of Human Creutzfeldt-Jakob Disease Using Cerebrospinal Fluid. MBio 6. 10.1128/mBio.02451-14 PMC4313917 25604790

[31] Orru CD, Hughson AG, Race B, Raymond GJ, Caughey B (2012) Time course of prion seeding activity in cerebrospinal fluid of scrapie-infected hamsters after intratongue and intracerebral inoculations. JClinMicrobiol 50: 1464–1466. 10.1128/JCM.06099-11 PMC3318555 22238438

[32] Orrú CD, Wilham JM, Hughson AG, Raymond LD, McNally KL, et al (2009) Human variant Creutzfeldt-Jakob disease and sheep scrapie PrP(res) detection using seeded conversion of recombinant prion protein. Protein Eng Des Sel 22: 515–521. 10.1093/protein/gzp031 19570812 PMC2719501

[33] Peden AH, Sarode DP, Mulholland CR, Barria MA, Ritchie DL, et al (2014) The prion protein protease sensitivity, stability and seeding activity in variably protease sensitive prionopathy brain tissue suggests molecular overlaps with sporadic Creutzfeldt-Jakob disease. Acta Neuropathol Commun 2: 152 10.1186/s40478-014-0152-4 25331173 PMC4210614

[34] Orru CD, Bongianni M, Tonoli G, Ferrari S, Hughson AG, et al (2014) A test for Creutzfeldt-Jakob disease using nasal brushings. New England Journal of Medicine 371: 519–529. 10.1056/NEJMoa1315200 25099576 PMC4186748

[35] Henderson DM, Manca M, Haley NJ, Denkers ND, Nalls AV, et al (2013) Rapid antemortem detection of CWD prions in deer saliva. PLoSONE 8: e74377 10.1371/journal.pone.0074377 24040235 PMC3770611

[36] Elder AM, Henderson DM, Nalls AV, Wilham JM, Caughey BW, et al (2013) In vitro detection of prionemia in TSE-infected cervids and hamsters. PLoS ONE 8: e80203 10.1371/journal.pone.0080203 24224043 PMC3815098

[37] Haley NJ, Carver S, Hoon-Hanks LL, Henderson DM, Davenport KA, et al (2014) Detection of chronic wasting disease in the lymph nodes of free-ranging cervids by real-time quaking-induced conversion. J Clin Microbiol 52: 3237–3243. 10.1128/JCM.01258-14 24958799 PMC4313144

[38] Kocisko DA, Come JH, Priola SA, Chesebro B, Raymond GJ, et al (1994) Cell-free formation of protease-resistant prion protein. Nature 370: 471–474. 7913989 10.1038/370471a0

[39] Kocisko DA, Priola SA, Raymond GJ, Chesebro B, Lansbury PT Jr., et al (1995) Species specificity in the cell-free conversion of prion protein to protease-resistant forms: a model for the scrapie species barrier. ProcNatlAcadSciUSA 92: 3923–3927. 10.1073/pnas.92.9.3923 PMC42074 7732006

[40] Raymond GJ, Hope J, Kocisko DA, Priola SA, Raymond LD, et al (1997) Molecular assessment of the transmissibilities of BSE and scrapie to humans. Nature 388: 285–288. 9230438 10.1038/40876

[41] Raymond GJ, Bossers A, Raymond LD, O'Rourke KI, McHolland LE, et al (2000) Evidence of a molecular barrier limiting susceptibility of humans, cattle and sheep to chronic wasting disease. EMBO J 19: 4425–4430. 10970836 10.1093/emboj/19.17.4425 PMC302048

[42] Bossers A, Belt PBGM, Raymond GJ, Caughey B, de Vries R, et al (1997) Scrapie susceptibility-linked polymorphisms modulate the *in vitro* conversion of sheep prion protein to protease-resistant forms. ProcNatlAcadSciUSA 94: 4931–4936. 10.1073/pnas.94.10.4931 PMC24608 9144167

[43] Priola SA, Chesebro B (1995) A single hamster amino acid blocks conversion to protease-resistant PrP in scrapie-infected mouse neuroblastoma cells. JVirol 69: 7754–7758. 7494285 10.1128/jvi.69.12.7754-7758.1995 PMC189717

[44] Goldmann W, Hunter N, Benson G, Foster JD, Hope J (1991) Different scrapie-associated fibril proteins (PrP) are encoded by lines of sheep selected for different alleles of the Sip gene. J Gen Virol 72 (Pt 10): 2411–2417. 1681027 10.1099/0022-1317-72-10-2411

[45] Groveman BR, Dolan MA, Taubner LM, Kraus A, Wickner RB, et al (2014) Parallel in-register intermolecular beta-sheet architectures for prion-seeded prion protein (PrP) amyloids. J Biol Chem 289: 24129–24142. 10.1074/jbc.M114.578344 25028516 PMC4148845

[46] Atarashi R, Moore RA, Sim VL, Hughson AG, Dorward DW, et al (2007) Ultrasensitive detection of scrapie prion protein using seeded conversion of recombinant prion protein. NatMethods 4: 645–650. 17643109 10.1038/nmeth1066

[47] Atarashi R, Wilham JM, Christensen L, Hughson AG, Moore RA, et al (2008) Simplified ultrasensitive prion detection by recombinant PrP conversion with shaking. NatMethods 5: 211–212. 10.1038/nmeth0308-211 18309304

[48] Saborio GP, Permanne B, Soto C (2001) Sensitive detection of pathological prion protein by cyclic amplification of protein misfolding. Nature 411: 810–813. 11459061 10.1038/35081095

[49] Groveman BR, Kraus A, Raymond LD, Dolan MA, Anson KJ, et al (2015) Charge neutralization of the central lysine cluster in prion protein (PrP) promotes PrP(Sc)-like folding of recombinant PrP amyloids. J Biol Chem 290: 1119–1128. 10.1074/jbc.M114.619627 25416779 PMC4294479

[50] Fox BG, Blommel PG (2009) Autoinduction of protein expression. Curr Protoc Protein Sci Chapter 5: Unit 5 23. 10.1002/0471140864.ps0523s56 19365792 PMC5602607

[51] Studier FW (2005) Protein production by auto-induction in high density shaking cultures. Protein ExprPurif 41: 207–234. 15915565 10.1016/j.pep.2005.01.016

[52] Caughey B, Raymond GJ, Ernst D, Race RE (1991) N-terminal truncation of the scrapie-associated form of PrP by lysosomal protease(s): implications regarding the site of conversion of PrP to the protease-resistant state. JVirol 65: 6597–6603. 1682507 10.1128/jvi.65.12.6597-6603.1991 PMC250721

