# Correction: Bank Vole Prion Protein As an Apparently Universal Substrate for RT-QuIC-Based Detection and Discrimination of Prion Strains

**DOI:** 10.1371/journal.ppat.1005117

**Published:** 2015-08-18

**Authors:** Christina D. Orrú, Bradley R. Groveman, Lynne D. Raymond, Andrew G. Hughson, Romolo Nonno, Wenquan Zou, Bernardino Ghetti, Pierluigi Gambetti, Byron Caughey

The following information is missing from the Funding section: BG is supported by NIH P30 AG10133.
